# Novel coronaviruses and mammarenaviruses of hedgehogs from Russia including the comparison of viral communities of hibernating and active specimens

**DOI:** 10.3389/fvets.2024.1486635

**Published:** 2024-12-16

**Authors:** A. V. Lukina-Gronskaya, I. K. Chudinov, E. V. Korneenko, S. D. Mashkova, T. A. Semashko, M. A. Sinkova, L. N. Penkin, E. M. Litvinova, N. Yu Feoktistova, A. S. Speranskaya

**Affiliations:** ^1^Laboratory of Multiomics Research, Scientific Research Institute for Systems Biology and Medicine, Federal Service on Consumer Rights Protection and Human Well-Being Surveillance, Moscow, Russia; ^2^Phystech School of Biological and Medical Physics, Moscow Institute of Physics and Technology, Dolgoprudny, Russia; ^3^Department of Epidemiology, Saint Petersburg Pasteur Institute, Federal Service on Consumer Rights Protection and Human Well-Being Surveillance, Saint Petersburg, Russia; ^4^Zoological Museum of Moscow State University Named After M.V. Lomonosov, Moscow, Russia; ^5^Biological Department, Lomonosov Moscow State University, Moscow, Russia; ^6^A.N. Severtsov Institute of Ecology and Evolution RAS, Moscow, Russia

**Keywords:** hedgehogs, *metavirome*, *Betacoronavirus*, Erinaceus coronavirus, Mammarenavirus, *novel viruses*, public health, *East Europe*

## Abstract

**Introduction:**

Small mammals, especially rodents and bats, are known reservoirs of zoonotic viruses, but little is known about the viromes of insectivorous species including hedgehogs (order Eulipotyphla), which often live near human settlements and come into contact with humans.

**Methods:**

We used high-throughput sequencing and metaviromic analysis to describe the viromes of 21 hedgehogs (Erinaceus sp.) sampled from summer 2022 to spring 2023. We captured 14 active animals from the wild (seven in European Russia and the other seven in Central Siberia). The remaining 7 animals were hibernating in captivity (captured in European Russia before the experiment).

**Results and discussion:**

The diversity of identified viral taxa as well as the total number of reads classified as viral was high in all active animals (up to eight different viral families per animal), but significantly lower in hibernating animals (zero or no more than three different viral families per animal). The present study reports, for the first time, betacoronaviruses and mammasrenaviruses in hedgehogs from Russia. Erinaceus coronaviruses (EriCoVs) were found in 4 of 7 active animals captured in the wild, in European Russia, making it is the easiest finding of EriCoVs in Europe. One animal was found to carry of two different EriCoVs. Both strains belong to the same phylogenetic clade as other coronaviruses from European hedgehogs. Pairwise comparative analysis suggested that one of these two strains arose by recombination with an unknown coronavirus, since all of identified SNPs (*n* = 288) were found only in the local genome region (the part of ORF1b and S gene). The novel mammarenaviruses (EriAreVs) were detected in 2 out of 7 active and in 2 out of 7 hibernating animals from the European Russia. Several complete L and S segments of EriAreVs were assembled. All identified EriAreVs belonged to the same clade as the recently described MEMV virus from Hungarian hedgehogs. As the hibernating hedgehogs were positive for EriAreVs when kept in controlled conditions without contact with each other, we suggest the possibility of persistent arenavirus infection in hedgehogs, but further experiments are needed to prove this.

## 1 Introduction

Small mammals, especially rodents and bats, are well-known reservoirs of zoonotic viruses (1, 2), but little is known about the virome of insectivore mammals. Hedgehogs (*Erinaceus*) (order *Eulipotyphla*) are insectivores that are solitary, territorial, non-migratory, hemisynanthropic with a variable range of habitats (3). Hedgehogs are occasionally found near garbage dumps and gardens in rural areas and small towns where they come into contact with humans. These small animals are increasingly kept as pets, and the danger of the hedgehog as a carrier of potentially zoonotic viruses is underestimated by the public. The importance of studying hedgehog-borne viruses, monitoring their spread and analyzing the potential threat these viruses pose to human health has been repeatedly emphasized. Hedgehogs are confirmed hosts of a tick-borne encephalitis virus (*Flaviviridae*) (4–6), rabies (*Rhabdoviridae*) and herpesviruses (4, 7, 8) that can affect human health. Multiple studies show that these animals can be carriers of viruses of the families *Coronaviridae* (9–16) and *Arenaviridae* (17, 18), on which this article will focus on. Coronaviruses of the genus *Betacoronavirus* occur worldwide and some of the viruses belonging to this genus are responsible for severe respiratory diseases in humans. The origin of the betacoronaviruses that infect humans is not fully understood, but phylogenetic analysis of viral genomes suggests that they originated in wild animals and were then transmitted to humans via intermediate hosts (19). There is no direct evidence that a hedgehog can be an intermediate host, but there are reasonable grounds for suspecting it (20). This explains the reason for studying coronaviruses from these animals. The first betacoronavirus, named EriCoV, was detected in hedgehogs (*E. europaeus*) captured in Germany in 2012 (9). Since then, EriCoVs have been described in France (14), Italy (10, 11), United Kingdom (16), Poland (15). Phylogenetically distinct betacoronaviruses have been reported in *E. amurensis* captured in China, in 2014 and 2019 (12, 13). This suggests that research on hedgehog coronaviruses living in Russia may lead to new discoveries.

Arenaviruses (genus *Mammarenavirus*) are of interest because several viruses of this genus, carried by small mammals, can infect humans and cause severe or fatal diseases (21–23). Most of what is known about arenaviruses in wild mammals comes from studies of bats (24) and rodents (mice, jerboas, rats) (17, 21, 22). Hedgehogs were not known to carry arenaviruses until 2023, when *Mecsek Mountains virus* (MEMV) was described in *E. roumanicus* in Hungary (18). Arenaviruses are expected to cause persistent infections in hedgehogs, similar to rodents (17). The zoonotic potential of hedgehog mammarenaviruses has not yet been confirmed, but they must be kept under surveillance, as rodent mammarenaviruses already pose a risk (25). For example, the neglected rodent-borne *Lymphocytic choriomeningitis virus* (LCMV) is distributed worldwide and it affects the human central nervous system sometimes seriously damaging the whole organism (22, 26).

In terms of virus ecology, it is important that hedgehogs are among those few mammals that actually hibernate. Hibernation is an energy-saving behavior that enables these animals to survive low temperature periods and is common to all European hedgehog species. There is evidence that hibernation (as well as the stress of awakening) may affect virus-host relationships in mammals, e.g., increased virus load of some viruses has been observed during hibernation of bats, such as gamma herpesvirus (EfHV) (27). To our knowledge, there is no published data on virus communities in hibernating hedgehogs, although this information could help to answer some questions, such as which viruses cause persistent infections in hedgehogs.

*Erinaceus europaeus* (Western European hedgehog) and *Erinaceus roumanicus* (Northern white-breasted hedgehog) are common species in European Russia and Western Siberia (prevalent throughout Europe and partly Asia, i.e., western Siberia and Kazakhstan) (28). There are two known zones of sympatry between *E. europaeus* and *E. roumanicus*: in central Europe (Poland, Czech Republic, Austria and Italy) and in northeastern Europe (Latvia, Estonia and European Russia east to the Urals). In the Eastern European sympatry zone, there is ongoing hybridization between *E. europaeus* and *E. roumanicus*, with the proportion of individual animals with mixed ancestry about 20% (29). Due to the possibility of hybrid origin of animals investigated here, we decided to designate the species affiliation of individual animals (not subjected to special zoological and molecular analysis) as *Erinaceus* sp.

As for viruses found in hedgehogs living in Russia, currently there is only little information based on real-time PCR and the indirect method of fluorescent antibody (MFA) (30, 31). The first aim of this study is to investigate the viral diversity and to describe the mammarenaviruses and betacoronaviruses of hedgehogs from European Russia and Siberia. The second aim of our work is to determine whether there is a difference in the virome composition between active and hibernating animals.

## 2 Materials and methods

### 2.1 Ethical approval

The methods employed for the capture and swabbing of animals in this study has been approved by the Ethics Committee of the Research Institute for Systems Biology and Medicine (RISBM), protocol number N1 (21/05/2024).

### 2.2 Sample collection and description

Twenty-one animals of the *Erinaceus* sp. (*N* = 21) were included in the study. The animals were caught by professional zoologists. The capture was done manually in the hedgehog’s natural habitat, in the evening, using hand-held flashlights and protective gloves. The hedgehogs were examined immediately at the point of capture. The zoologists then waited for the animals to uncurl, after which they swabbed the animals and released them into their natural habitat. In total, 43% (9/21) of the collected animals were females and 57% (12/21) were males. The animals were captured between summer 2022 and spring 2023 in different regions of Russia, in urban or suburban areas of cities. The geographic interposition of sampling locations, IDs of samples and other metadata are presented in Supplementary Table S1 “Sample Description.” There were fourteen active hedgehogs, captured in the wild (seven in European Russia and the rest in Siberia) during this investigation. Oral and anal swabs were taken from each of the active animals, after which they were immediately released into their natural habitat unharmed.

Seven hibernating hedgehogs were provided by zoologists from the Zoological Museum of the Lomonosov Moscow State University. The hedgehogs had been rescued by zoologists and subsequently transferred to the Zoological Museum for the rehabilitation. The animals were admitted for rehabilitation between 10.08.22 and 07.10.22. Most of them had signs of illness (suspected pneumonia) and underweight. All these animals had been kept in separate cages and had had no contact with each other before sampling. Swabs were taken during hibernation, approximately 1 month after the start of hibernation, on the same day in all animals, 21.11.22. For bioethical reasons, only oral swabs were taken from temporary awaking animals: because the hedgehogs were awakened during hibernation, they were weak. It was decided that there was too great a risk in forcibly uncurling them or waiting for them to fully awaken, as the animals might not return to hibernation, their habitual lifestyle would be disrupted, and further adaptation to wild conditions would be impossible. In spring 2023, animals were released into their natural habitat, while others stayed in the museum due to their inability to survive in the wild.

The swabs were placed in a mucolytic transport medium (AmpliSens, Russia) and kept at 4°C before transported to the laboratory. Prior to laboratory processing, the samples were stored at −70°C.

### 2.3 RNA extraction

RNA was extracted from oral and anal swabs using the QIAamp viral RNA mini kit (Qiagen, Germany), then the RNA extracts were treated with NEB DNase I (RNase-free) (NEB, USA) according to the manufacturer’s instructions. The 140 μL of resuspended fecal or oral samples were used for a start. Further steps were performed according to the original protocol. Due to the ineffective action of DNAase, the residual DNA remained in the resulting nucleic acid extracts. A total of 10 μL of RNA/DNA mixture was taken for the next step.

### 2.4 Library preparation and high-throughput sequencing

The first strand of cDNA was obtained using the NEBNext Ultra II RNA First Strand Synthesis Module (NEB, USA), and the second strand of cDNA was obtained using the NEBNext UltraII Non-Directional RNA Second Strand Synthesis Module (NEB, USA). End preparation was performed using the NEBNext End Repair Module, which is part of the NEBNext Ultra II DNA Library Prep Kit for Illumina (NEB, USA). MGIEasy DNA Adapters (MGI, China) in the amount of 1.25 μL were ligated to double-stranded cDNA using a ligation module which is also part of the NEBNext Ultra II DNA Library Prep Kit for Illumina (NEB, USA). The libraries were then amplified using PCR Primer Mix and PCR Enzyme Mix from the MGIEasy FS DNA Library Prep Kit (MGI, China).

High-throughput sequencing was performed using the DNBSEQ-G400RS High-throughput Rapid Sequencing Kit (FCS PE100 and FCL PE150) and the DNBSEQ-G400RS Rapid Sequencing Flow Cell.

To confirm the presence of two strains in one sample, the sample with ID 22_15(MOS)-o was double sequenced on the Oxford Nanopore Technologies (ONT) platform (in addition to MGI sequencing). For ONT library preparation, end preparation was performed using the NEBNext Ultra II End Repair/dA-Tailing Module (NEB, USA). Index ligation was performed using Blunt/TA Ligase Master Mix. Libraries were then amplified using the PCR Barcoding Extension (EXP-PBC096). High-throughput sequencing was performed with GRIDIon Oxford Nanopore Technologies platform using the SpotON Flow Cell (R9.4) and Flow Cell Priming Kit (EXP-FLP002).

### 2.5 Detecting NGS data sets for virus reads

The quality control of raw reads was performed using FastQC v. 0.11.9 (32). The reads were filtered using Trimmomatic v. 0.39 (33) with default parameters. The sequences of MGI adapters were trimmed. As our laboratory works with clinical samples containing human SARS-CoV-2, the obtained sequence data were additionally checked by bioinformatic methods and the samples were additionally checked by targeted PCR methods for the presence of SARS-CoV-2 contamination. Raw reads from the severe acute respiratory syndrome coronavirus 2 isolate Wuhan-Hu-1 were mapped to complete genome reads using alignment to NC_045512.2 and then were removed. The purified reads were then classified using kraken2 against the custom database, which consists of the kraken2 databases (database assembly 01.12.2023) (34). The resulting read count statistics were used to extract all Nroot values of F-ranked entries (corresponding to virus families). In the kraken2 documentation, this information is described as the number of fragments covered by the clade rooted at that taxon. In this work, we evaluated number of reads referred to specific clade as the representation of the viral family in terms of “reads placement count” metrics neither than taxon abundance.

Data post-processing included the following steps: (a) removal of all family columns where reads for any sample were 20 or less; (b) normalization to the total number of reads; (c) the resulting relative number of reads is multiplied by 1,000,000 so that we can talk in parts per million reads, (ppm); (d) values are visualized, color scale is logarithmic, values are formatted to the tenth.

To confirm and validate the results obtained by kraken2, we used the Genome Detective Viral Tool (35). Where discrepancies occurred, the reads were subjected to additional manual analysis by mapping the raw reads to reference virus sequences from the target family.

### 2.6 Betacoronaviruses and arenaviruses complete genomes assembly

**Figure 1:**
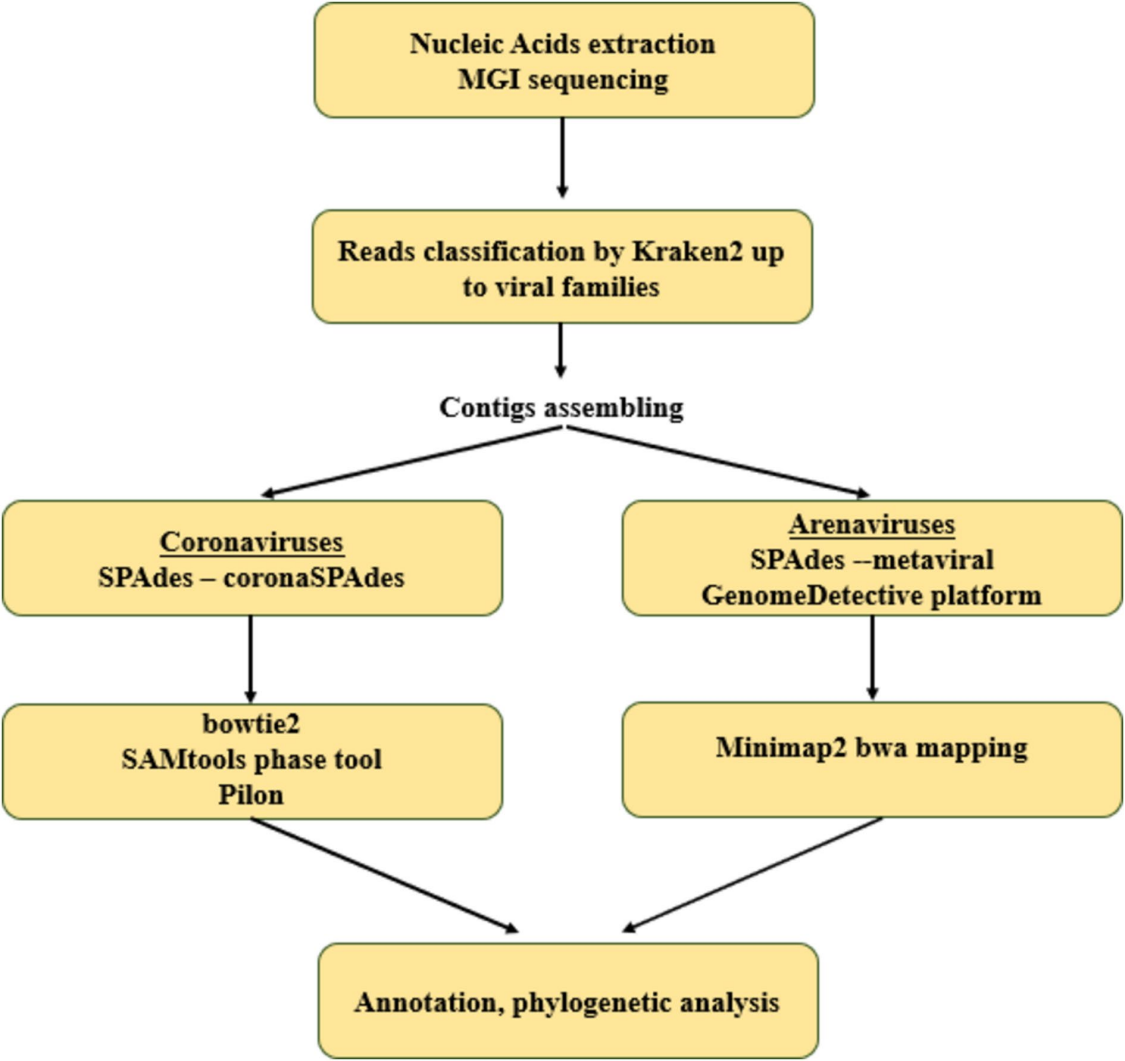
The scheme of pipeline for coronaviruses and arenaviruses genomes assembling.

We performed *de novo* assembly of reads obtained from positive animals using hybrid approaches, different for *Coronaviridae* and *Arenaviridae* (schematically depicted in Figure 1).

#### 2.6.1 Betacoronavirus complete genome assembly

The consensus genome assembly of betacoronaviruses was performed using the coronaSPAdes pipeline in SPAdes v. 3.15.5 (36). The reads were mapped to the assembled genome using bowtie2 v. 2.5.3 (37) with default parameters. The diversification of the consensus sequence into 2 homologous genomes was performed using the Samtools phase tool in SAMtools 1.13 (38) and pilon 1.20 with -fix bases parameter (39). Some of the above procedures were performed on the Galaxy platform (40).

#### 2.6.2 Complete genome assembly of arenaviruses

Metagenomic assembly was performed using the metaviralSPAdes pipeline in SPAdes v3.13.1 (41). Mammarenavirus contigs were identified using minimap2 v. 2.26-r1175 (42) by alignment to a database consisting of all mammarenavirus sequences from Genbank (accessed 20.03.2024). Further reads were aligned to the resulting sequences using the BWA-MEM algorithm in BWA software v. 0.7.18 (43). According to the obtained alignments, the assemblies were corrected by removing low coverage regions (<10 nt). Next, the assemblies were evaluated in the web server of the multiple alignment program for amino acid or nucleotide sequences v. 7 (44) and corrected manually if necessary.

### 2.7 Assembled genome annotation

For the assembled coronavirus genomes, ORFs were predicted using the Open Reading Frame Finder NCBI,^1^ blastn and blastx (45). Theprediction of protein domains was performed using InterProScan (46) and prokka command line tool v. 1.14.6 (47). The predicted CDS and protein domain were manually corrected.

### 2.8 Phylogenetic analysis

Alignments were performed using the Multiple Sequence Alignment (MSA) tool, MAFFT v7.505 (2022/Apr/10) (44), using default parameters. Phylogenetic analyses, including determination of the best-fitting nucleotide substitution model, were performed using W-IQ-TREE [multicore version 2.2.2.3] with ModelFinder (48), tree reconstruction (49) and ultrafast bootstrap (1,000 replicates) (50). The resulting tree topology was visualized and annotated using ITOL, online version (51).

For the phylogenetic analysis of coronaviruses, we downloaded the complete genome sequences belonging to the subgenus *Betacoronavirus* of 28,000–33,000 bp from NCBI GenBank. The downloaded sequences were filtered for duplicates, then SARS-CoV and SARS-CoV-2 sequences were excluded from sampling, except for SARS [AY274119] and SARS-CoV-2 [MN908947]. The maximum likelihood (ML) phylogenetic tree was generated with bootstrap (1000) for the coronavirus genome sequences assembled in this study and 1,556 sequences from NCBI. For IDs of complete genome sequences used, see Supplementary Table S2 “Coronaviruses Complete genomes ID.” Additionally, we examined the phylogenetic relationship of coronaviruses using all available public partial *RdRp* sequences (⁓500 bp). For the list of partial *RdRp* sequences used, downloaded from NCBI, see Supplementary Table S3 “Coronaviruses *RdRp* ID”.

For the phylogenetic analysis of arenaviruses, the *RdRp* gene of six assembled L-segments and NP genes of five assembled S-segments were aligned with homologous sequences of closely related viruses available in GenBank. For the list of analysis sequences, see Supplementary Table S4 “Arena L- and S-segments ID.”

### 2.9 Confirmation of the presence of two betacoronaviruses in the hedgehog ID 22-15(MOS)

The presence of two genomes in the sample with ID 22-15(MOS) was confirmed by sequencing on the Oxford Nanopore platform. Quality control of the reads was performed using NanoPlot v. 1.42.0 (52). Low quality (<8) and short length (<220 nt) raw reads were filtered using Chopper v. 0.7.0. The filtered reads were mapped to the assembled genomes using minimap2 v. 2.26-r1175 (42). Based on the resulting alignments, reference-based assemblies were obtained using samtools-consensus in SAMtools v. 1.19.2 (38) for draft genome construction and medaka-consensus for polished genome.

The two assembled genome sequences and four other sequences related to the subgenus *Merbecovirus* from the ICTV database were aligned using MAFFT v7.505 (44). Similarity plots were generated using SimPlot++ (53) with a sliding window of 400 and a step size of 40 nucleotides.

### 2.10 Data availability

All the complete and partial genome sequences were submitted to GenBank. The accession numbers for coronaviruses sequences are PP421220 and PP421221 (two genome sequences from animal ID 22-15(MOS)) and short contig sequences from animal ID 22-11(KRA) presented in Supplementary Table S5 “EriCoVs assembly.” The accession numbers for arenavirus L segments are PQ041964, PQ041965, PQ059273, PQ059276-PQ059278 and for S segments PQ041966, PQ041967, PQ059274, PQ059275, PQ059279.

The MGI raw sequence data and ONT reads obtained for the hedgehog ID 22_15 (MOS) were deposited in the NCBI SRA database under accession number PRJNA1103355.

## 3 Results

### 3.1 Metaviromic analysis of hedgehog’s oral and anal swabs

**Figure 2:**
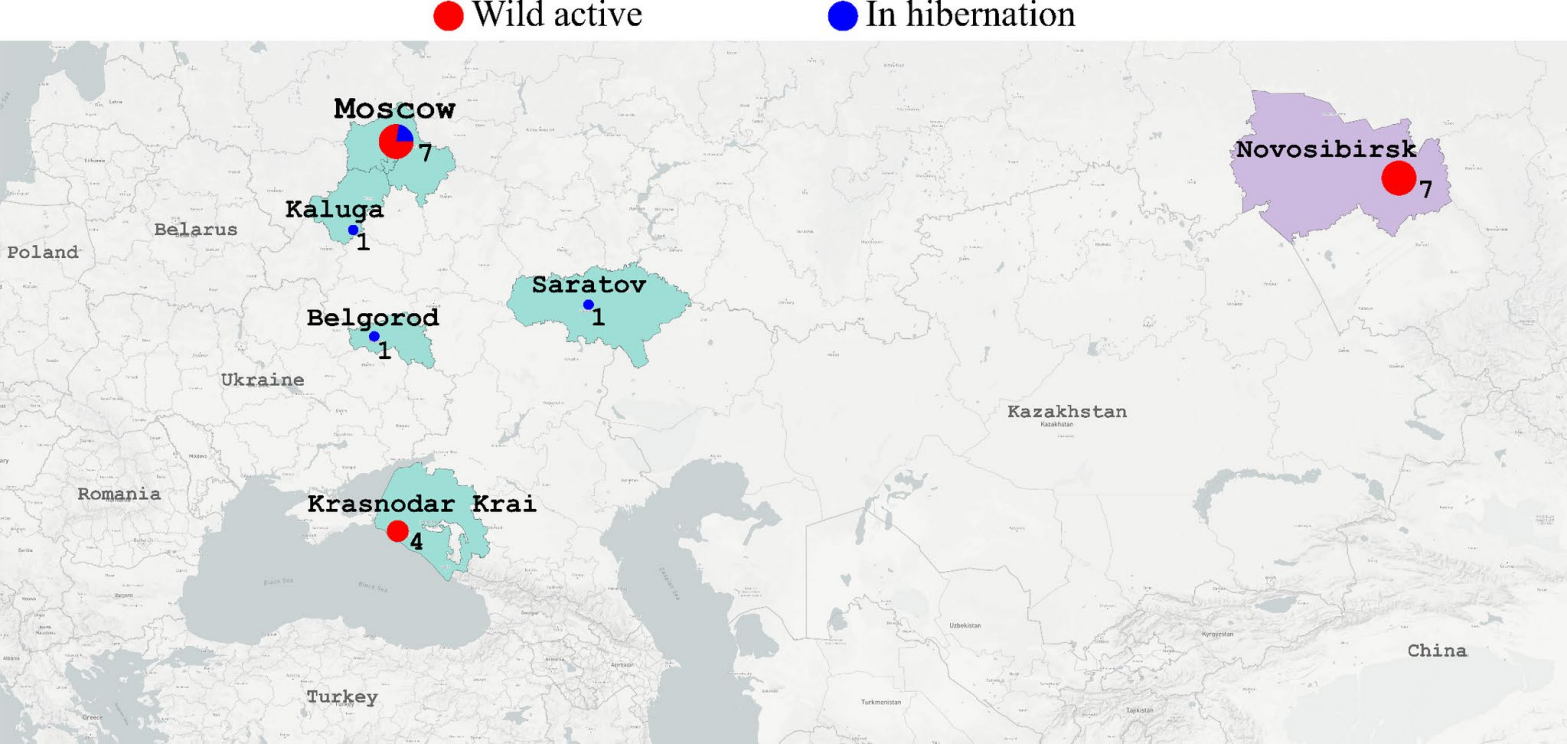
The map shows where the hedgehogs were captured. European regions of Russia (Moscow, Kaluga, Belgorod, Saratov, Krasnodar Krai) are colored in light green. Siberian region (Novosibirsk) is colored in light lilac. Red circles - active hedgehogs sampled to obtain the swabs immediately. Blue circles - hedgehogs which were hibernating in the rehabilitation center (swabs were collected during hibernation). Diameter of circles reflects number of samples collected in the region.

All the twenty-one animals of *Erinaceus* sp. were captured in Russia. Out of fourteen active animals all of which were captured in the wild, half come from European Russia (*n* = 7) and the remaining part from Central Siberia (*n* = 7). From the active animals, two types of samples, oral and anal, were collected. The seven hibernating animals originated from European Russia. From the hibernating animals, only oral swabs were collected (Figure 2).

**Figure 3:**
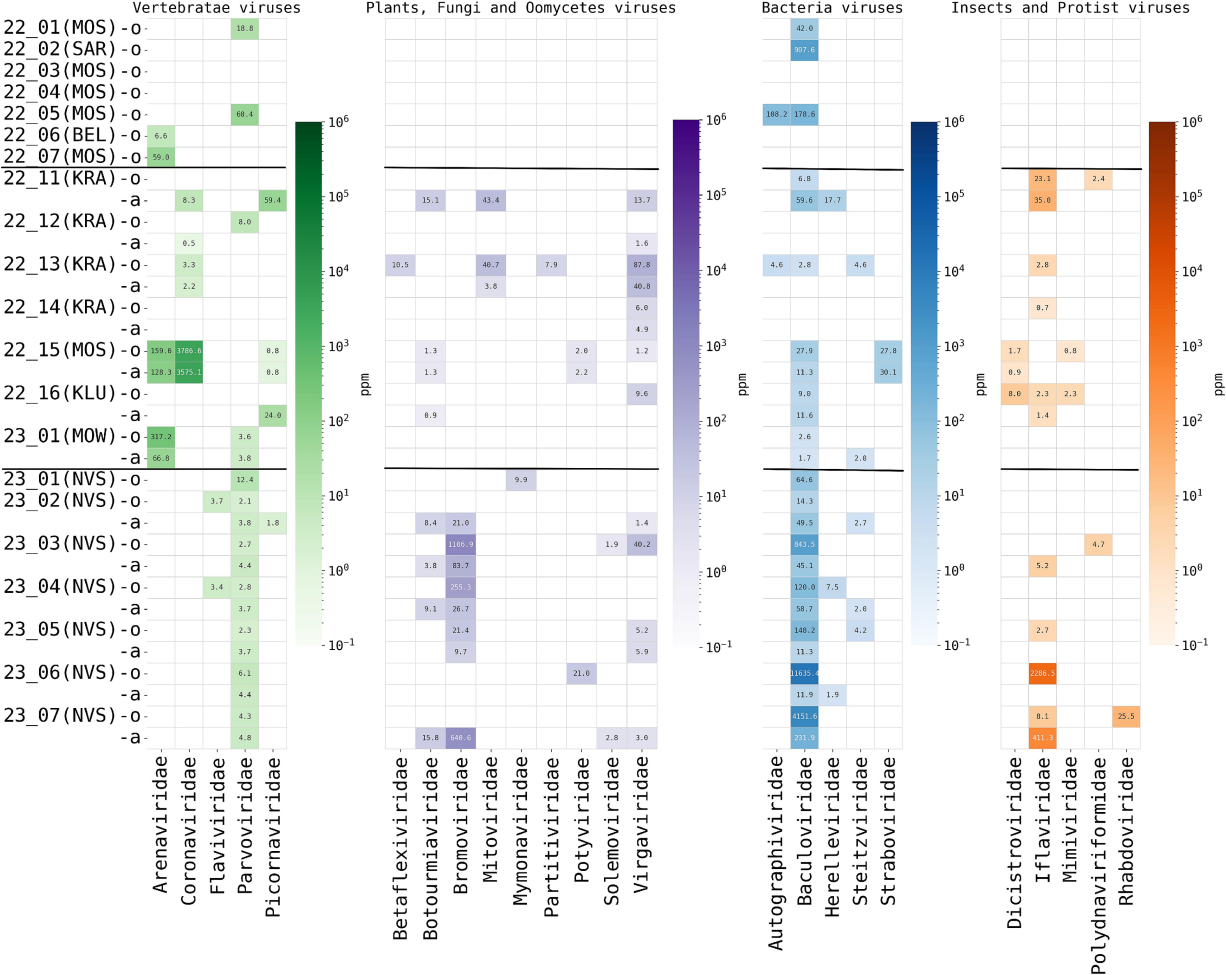
Read-based taxonomic profiling of hedgehog metaviromes using Kraken2 (threshold—20 reads) from oral and anal swabs analyzed by high-throughput total RNA sequencing. The abundance of viruses in individuals is expressed as the number of Kraken2 classified reads per million total reads (ppm). The heatmap shows the distribution and abundance of vertebrate-associated viruses in the sequence data of the hedgehogs studied herein. Each column represents a virus family, while each row represents an individual animal caught in the region (in brackets) for oral (prefix -o) and anal (prefix -a) swabs. Horizontal stripes separate animals captured in different regions of Russia, from top to bottom: European Russia (hibernating animals in captivity), European Russia (active animals, in the wild), Central Siberia (active animals, in the wild).

High-throughput sequencing and bioinformatic analysis of nucleic acids extracted from oral and anal swabs was performed to determine the presence of viral reads. A total of 24 different viral families were detected in the sequence data using the kraken2 taxonomy identification methods. Viral putative host predictions were made using the taxonomy defined by kraken2 and according to the ICTV (International Committee on Taxonomy of Viruses) description of viral families with additional manual analysis. For example, reads from *Rhabdoviridae* were identified in the hedgehog ID 23_7(NSV). We assembled the complete rhabdovirus genome, but further analysis showed that the closest relatives of this virus are the rhabdoviruses infecting insects. The resulting taxonomic definition of the identified viral nucleic acids is presented in Figure 3. For details, the absolute number of reads classified as belonging to one or another viral family obtained by kraken.2 is shown in Supplementary Table S6 “Kraken2ViralReadsCount” and obtained by the Genome Detective Viral Tool, presented in Supplementary Table S7 “Genome Detective Coronaviruses” and Supplementary Table S8 “Genome Detective Arenaviruses.” The most commonly identified viruses were putative vertebrate viruses (5/24 of identified virus families), plant/fungal viruses (9/24), insect/invertebrate viruses/amoeba/protists (9/24) and bacteriophages (5/24).

**Figure 4:**
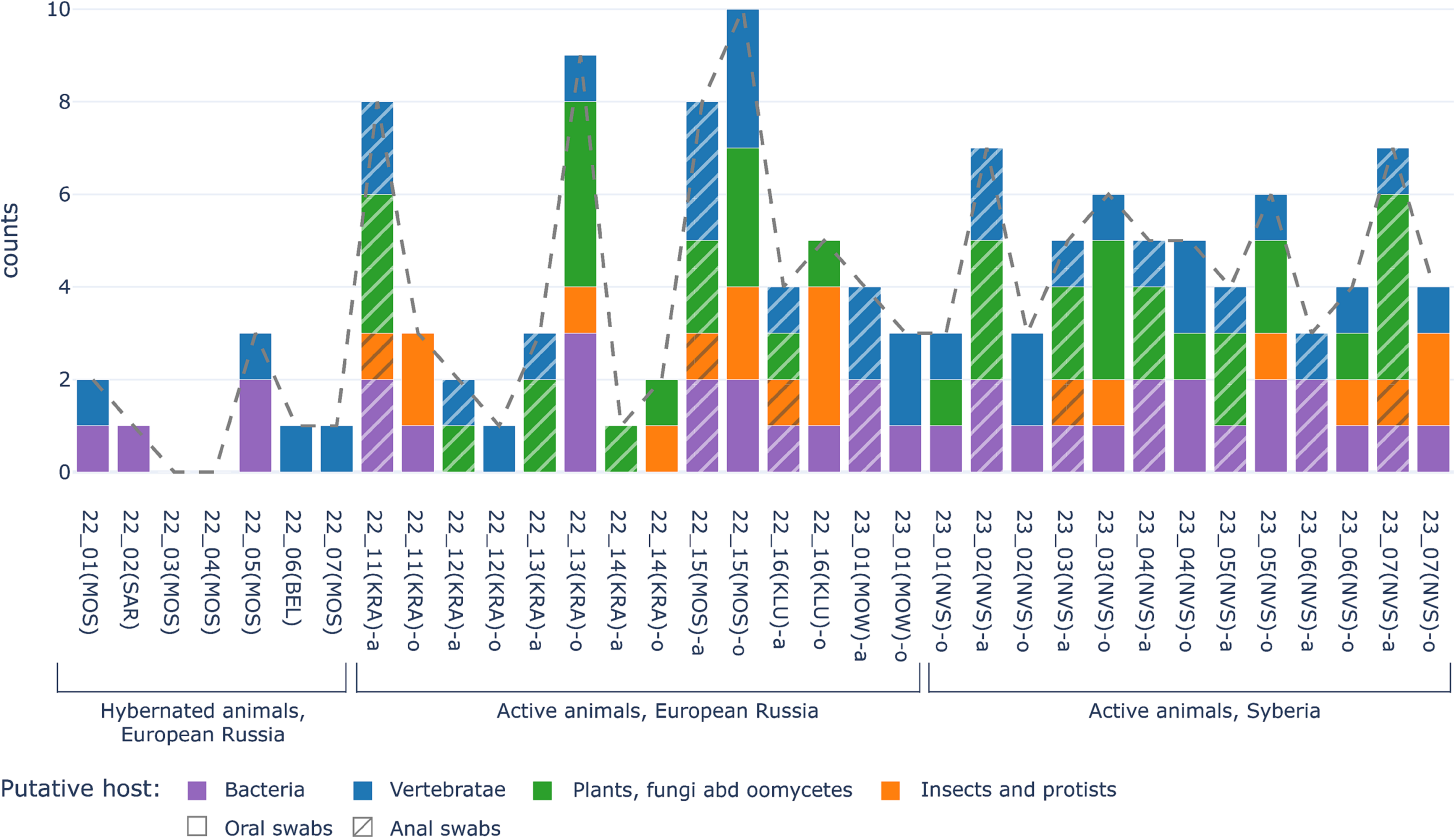
Comparison of numbers of viral families identified in swabs from hibernating animals (left part of diagramm) and from active hedgehogs in the wild (central part of diagramm - for European Russia, right part of diagramm—for Central Siberia). X-axis shows sample ID. The Y-axis is the number of families identified. Putative viruses of vertebrates are marked by blue, putative viruses of plants, fungi and oomycetes are green, putative viruses of insects and protists are orange, bacteriophages are marked violet, while the dotted line shows total number of eukaryotic virus families.

The total amount of viral taxa detected was high in all active animals, up to eight different viral families per animal (Figure 4). However, the composition of virome was different for hedgehogs from different regions (European Russia and Central Siberia). For putative mammalian viruses, *Parvoviridae* reads were found in all oral and anal samples of all animals from Siberia (7/7), while in active animals from European Russia they were found in 2 of 7 animals. *Flaviviridae* reads were found only in Siberian animals (2/7). *Arenaviridae* (genus *Mammarenavirus*) and *Coronaviridae* (genus *Betacoronavirus*) reads were found only in animals from European Russia, in 2/7 and 4/7 of active hedgehogs, respectively. Plant and other non-mammalian viruses, such as *Bromoviridae* (plants are the putative hosts), were identified only in hedgehogs from Siberia, while *Dicistroviridae* (viruses of invertebrates) and *Mimiviridae* (amoebae and protists are the putative hosts) were identified only in hedgehogs from European Russia.

The main difference between viromes from active and hibernated animals concerned the total number of viral sequences (reads) and identified viral taxons, ranging from up to 6 different viral families (up to 3,786,6 ppm reads per family) in oral swabs of active animals, but no more than 3 different viral families (no more than 1,000 ppm reads per family) in oral swabs of hibernating animals. Overall, only four out of seven samples from hibernating animals contained any viral nucleic acids, representing one to three different virus families, as shown in Figure 4. No reads were identified that were predicted to be of origin of plant, fungal, oomycetes, insect or protist viruses were found in hibernating hedgehogs, however, putative mammalian and bacterial virus-related sequences were found. The two hibernating animals were positive for *Arenaviridae* and two others for Parvoviridae. Sequences of bacteriophages were found in three out of seven animals (Figure 3). Overall, although statistical methods are not applicable (due to the small sample size), viral diversity seems to correlate with the physiological status of the animals. This observation seems logical, since hibernating animals were hungry and therefore should not contain viruses from their food (i.e., insect viruses, viruses from plants that are food for insects, etc.)”

### 3.2 Detection and analysis of coronaviruses

In total, four animals were found positive for Erinaceus coronaviruses (another 3 samples were considered contaminated with nucleic acids of SARS-CoV-2 due to regular works in our laboratory). One of EriCoV-positive animals was caught in the city Moscow and three animals were caught in the Krasnodar Region (the distance between these geographical points is ~1,500 km). In total, coronaviruses were found in 4/7 animals caught in the wild of the European part of Russia (the Moscow Region and the Krasnodar Region). However, zero (0/7) of EriCoV-positive animals were found in Siberia. No reads classified as coronaviruses were found in hibernating hedgehogs (Table 1).

**Table 1:** The description of high-throughput sequencing datasets and Coronaviruses and Arenaviruses sequences assembled.

Sample ID	Oral swabs (−o)	Anal swabs (−a)	Coronaviruses (NCBI ID)	Arenaviruses (NCBI ID)
European part of Russia, active animals, in wild				
23_1(MOW)	28,103,912	24,321,164	–	L, PQ059278
				S, PQ059279
22_15(MOS)	79,907,216	79,472,076	PP421220, PP421221	L, PQ059276
				L, PQ059277
				S, PQ041967
				S, PQ059275
22_16(KLU)	19,475,230	56,618,474	–	–
22_11(KRA)	19,843,514	16,931,724	7 short contigs	–
22_12(KRA)	15,045,244	94,215,656	Positive, sequence not assembled	–
22_13(KRA)	27,445,084	48,947,046	Positive, sequence not assembled	–
22_14(KRA)	67,350,200	72,741,030	–	–
Siberia, active animals, in wild				
23_1(NVS)	7,094,690	NA	–	–
23_2(NVS)	40,332,574	29,164,920	–	–
23_3(NVS)	31,281,692	22,144,514	–	–
23_4(NVS)	24,711,646	24,683,434	–	–
23_5(NVS)	26,106,496	25,327,214	–	–
23_6(NVS)	34,143,082	25,900,874	–	–
23_7(NVS)	15,909,402	21,197,118	–	–
European Russia, hibernating animals, in captivity				
22_1(MOS)	5,001,058	NA	–	–
22_3(MOS)	23,892,196	NA	–	–
22_4(MOS)	17,135,658	NA	–	–
22_5(MOS)	893,260	NA	–	–
22_7(MOS)	20,625,774	NA	–	L, PQ059273
				S, PQ059274
22_2(SAR)	497,664	NA	–	–
22_6(BEL)	15,657,882	NA	–	L, PQ041964
				L, PQ041965
				S, PQ041966

For hedgehogs IDs 22_11(KRA), 22_12(KRA), 22_13(KRA), the number of coronaviruses reads varied from 25 to 100 per sample. We suppose these hedgehogs were carriers of coronaviruses, but the read frequency was too low for assembling. Using the KC545383.1 as references, we could assemble the short contigs only for one animal ID 22_11(KRA). The eight short contigs of 200–315 nt length with average read coverage of 4–10 represented betacoronavirus genome fragments with a total of 1,648 nt (~5,46% of the 30,826 nt reference genome ErinaceusCoV/Italy/50265–11/2019 [MW245799]). The genome contigs were not deposited in GenBank, but they are presented in Supplementary Table S5 “EriCoVs assembly.” The results of contig mapping to the reference genome (MW245799) are presented in Supplementary Figure S1.

The hedgehog ID 22-15(MOS) had high reads classified as *Coronaviridae* (genus *Betacoronavirus*), with 142,054 (0.36%) and 145,604 (0.38%) reads identified from oral and anal swabs, respectively. The two contigs of 30,315 bases, i.e., 99.99–100% of expected 30 kb for *Coronaviridae* were assembled and interpreted as genome sequences of two different strains from the same animal. These results were confirmed by double sequencing on the MGI and Oxford Nanopore Technologies (ONT).

The two complete betacoronavirus genomes from the oral swab of hedgehog ID 22-15(MOS) were named EriCoV/RU/MOW15-1/2022 and EriCoV/RU/MOW15-2/2022 (GenBank PP421220 and PP421221), in short EriCoV/MOW15-1 and EriCoV/MOW15-1. The genomes of EriCoV/MOW15-1 and EriCoV/MOW15-2 pairwise alignment showed that they have 98.87% identity and differ by 342 nucleotides, all of which are in the region of 13,444–22,478 nt. The genome organization of both EriCoVs variants assembled from hedgehog ID 22_15(MOS) was similar to other members of betacoronavirus genus and included 10 CDS: ORF1ab, spike (S), ORF3a, ORF3b, ORF4a, ORF4b, ORF5, envelope (E), membrane (M), nucleocapsid (N) and ORF8b: the details are presented in Supplementary Table S9 “EriCoVs ORF annotation”. Each virus genetic variant was analysed for coronavirus polyprotein (ORF1ab) which contains overlapping open reading frames encoding polyproteins. These polyproteins are cleaved to produce 16 non-structural proteins, Nsp1-16, for details see Supplementary Table S10 “EriCoVs Nsp annotation.”

**Figure 5:**
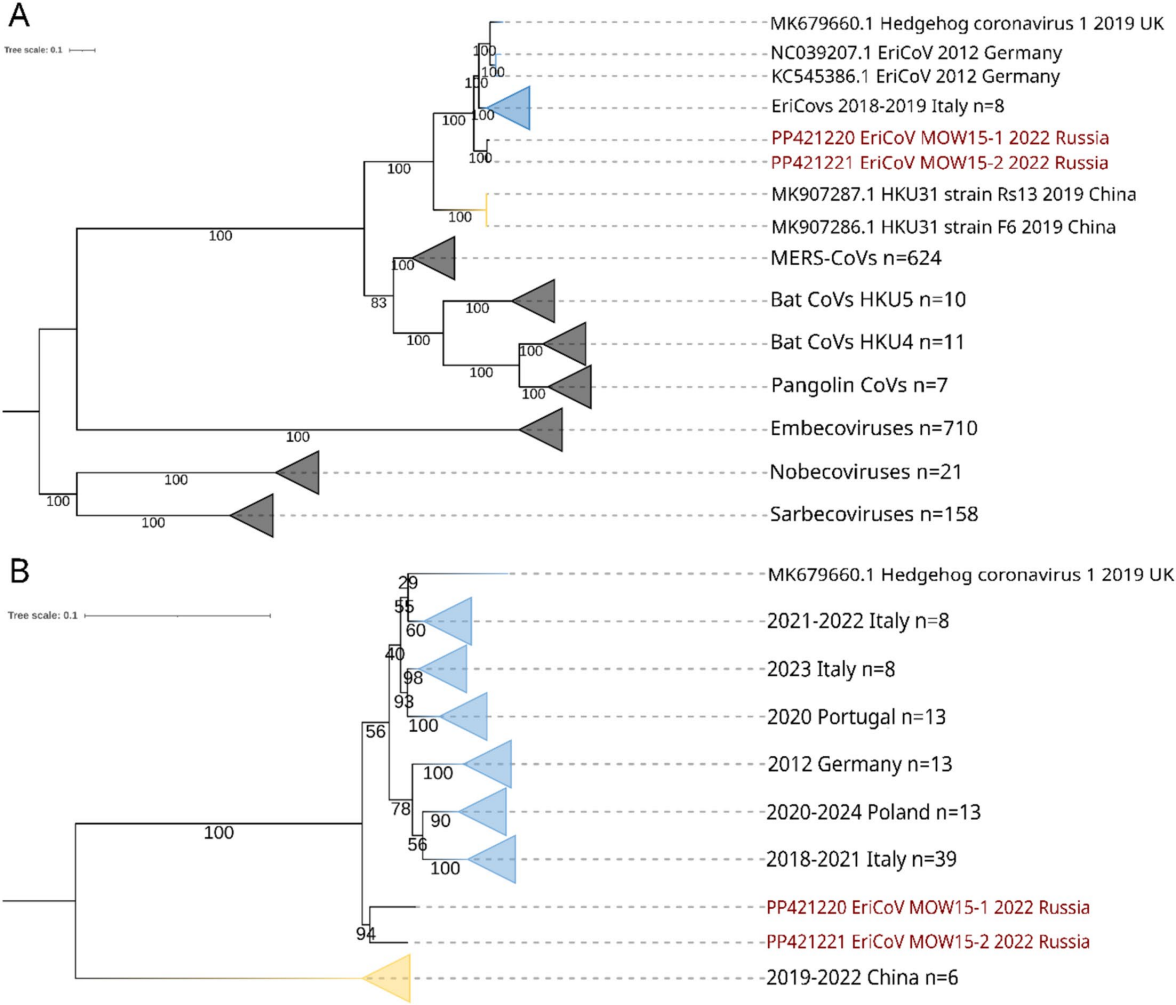
ML phylogenetic trees constructed for betacoronavirus sequences. Numbers indicate bootstrap values. The two viruses, EriCoV/RU/MOW15-1/2022 and EriCoV/RU/MOW15-2/2022, assembled in this study form the same hedgehog ID 22_15(MOS) are labelled in red. Yellow collapsed branches are for viruses from China, while blue collapsed branches are for viruses from Europe (Germany, Italy, Poland, UK, Portugal). **(A)** Tree constructed for complete genomes of different members of *Coronaviridae*. Best-fit substitution model according to BIC: GTR + F + I + G4; **(B)** Tree constructed for 91 partial *RdRp* gene sequences (~500 nt) of erinaceus coronaviruses. Best-fit model of substitution according to BIC: TIM3 + F + I + G4.

According to BLASTn search and phylogenetic analysis, EriCoV/MOW15-1 and EriCoV/MOW15-2 belong to the subgenus *Merbecovirus*, clade EriCoVs. In concordance with phylogenetic analysis of complete genome sequences, they are located between EriCoVs from Western European hedgehogs and HKU31 from Chinese hedgehogs, but represent an outgroup relative to all viruses from Western European hedgehogs, see Figure 5A. It should be noted that the assembled genomes of these two new variants EriCoVs from Russia (2022) were genetically closer to viruses from distant Italy (2018–2019) than to viruses from proximal Germany (2012). That means that phylogenetic proximity does not correlate with geographic distance, as might be expected since hedgehogs are non-migratory territorial animals. However, this proximity correlates with the date of biomaterial collection.

Additionally, we analyzed hedgehog coronaviruses using short *RdRp* gene fragments (500 bp). The 91 short *RdRp* sequences from 6 countries were obtained from NCBI and used for phylogenetic analysis, together with sequences obtained in this work. We found, both EriCoVs from Moscow hedgehog ID 22_15(MOS) is located on phylogenetic tree between Western European and Chinese EriCoVs, representing an outgroup to all viruses from Western Europe. In the clade of viruses from Western European hedgehogs, the bootstrap values were very low, and all viral sequences are intermingled in subclades with no separation by region or data, see Figure 5B.

**Figure 6:**
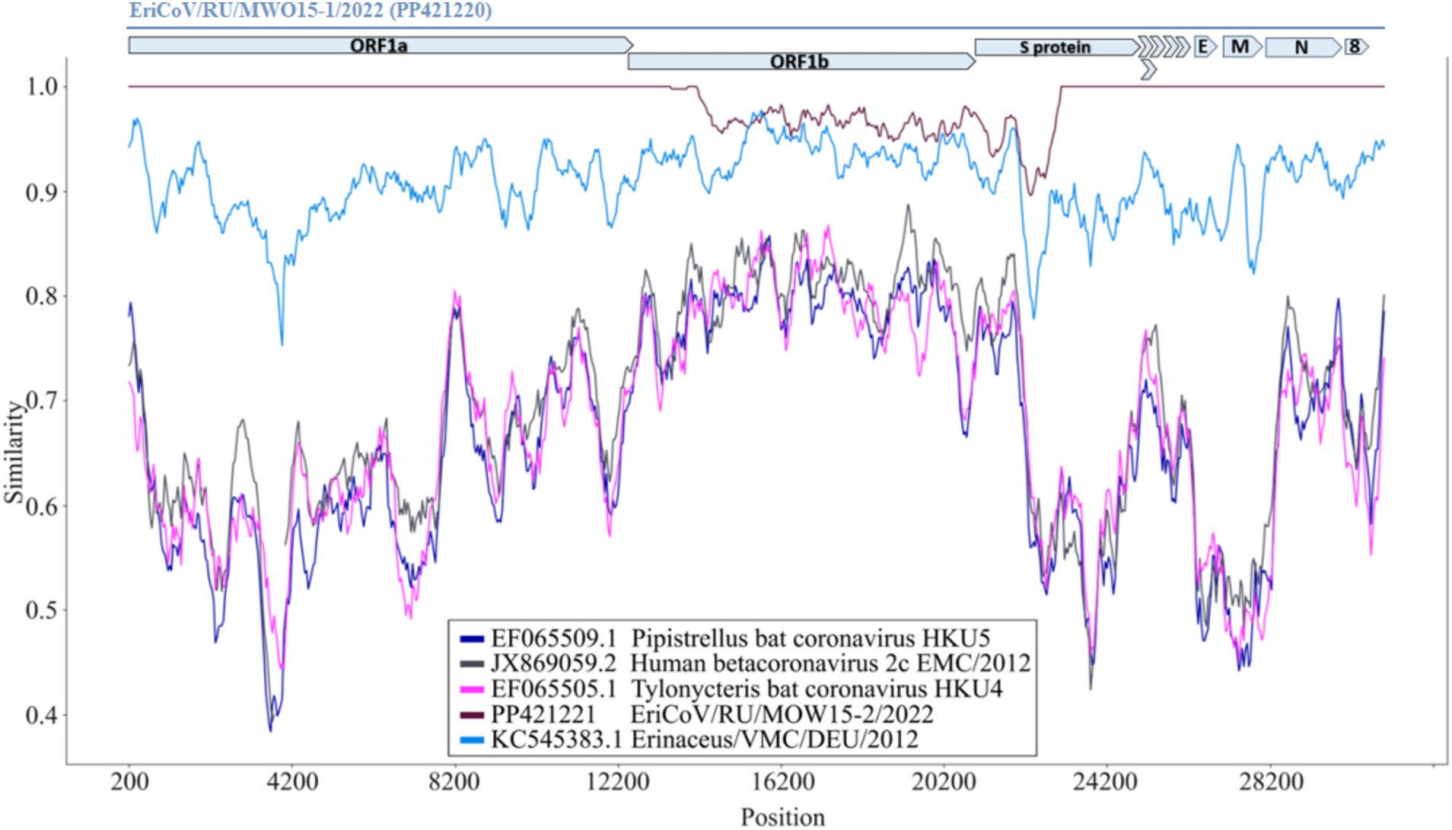
Similarity plot for EriCoV/MOW15-1 and EriCoV/MOW15-2, investigated here, first of known EriCoV genome from Germany and three other members of *Merbecovirus* subgenus. Parameters: window: 400 bp, step 40 bp, model: percent. The EriCoV/MOW15-1 used as reference and depicted by grey colored ORF and gene boxes (above the plot). Brown line—EriCoV/MOW15-2 (investigated here). Light-blue line - EriCoV from the hedgehog from Germany (KC545383.1). Pink line - HKU4 coronavirus from a Tylonycteris bat (EF065505.1). Dark-blue line—HKU5 coronavirus from a Pipistrellus bat (EF065509.1). Black line—MERS virus from human (JX869059.2).

To analyze the region with mutations in the genomes of other MERS-like betacoronaviruses comparative analysis of genomes assembled in this work and published previously was performed. The two genomes assembled in this work (EriCoV/MOW15-1 and EriCoV/MOW15-2) – were compared with several genomes of members of the subgenus *Merbecovirus*, including hedgehog betacoronavirus from Germany (KC545383.1), two bat betacoronaviruses, HKU4 (EF065505.1) and HKU5 (EF065509.1), and the human MERS virus (JX869059.2). The complete genome of EriCoV/MOW15-1 was used as the reference sequence, while the complete genomes of the other coronaviruses were scanned for SNP using a sliding window approach. We found that the most conservative region of the compared coronavirus genomes contained the most part of ORF1b and the 5′- region of the S gene. This is best illustrated by comparing plots of three non-hedgehog virus genomes (bat viruses HKU4 and HKU5 and human MERS) with three hedgehog viruses (MOW15-1, MOW15-2 from Russia and EriCoV genome from Germany) in Figure 6. Surprisingly, we found that the genomes of MOW15-1 and MOW15-2 differ from each other in these conserved regions, i.e., all identified SNPs are located in the ORF1b (288 SNP, coordinates 13,444–21,892 nt,) and in the S gene (54 SNP, coordinates 21,910–22,478 nt). The list of SNPs is presented in Supplementary Table S11 “EriCoVs List of SNPs”. Notable, no SNP were found in the ORF3-ORF8, E, M or N genes during the genome comparison of MOW15-1 and MOW15-2 viruses. But a number of SNP were found between these two genomes from Russian hedgehog and genome of betacoronavirus EriCoV from Germany hedgehog (KC545383.1). We hypothesize that the observed phenomenon (of completely coincidence of 5′- and 3′- parts and pronounced difference in the middle of genomes of two betacoronaviruses sequenced, assembled and confirmed from one animal) could be explained by recombination of two unknown ancestral betacoronaviruses.

### 3.3 Detection and analysis of novel Mammarenaviruses

Reads classified as *Arenaviridae* (genus *Mammarenavirus*) were detected in four examined animals, all captured in European Russia. The number of mammarenavirus reads ranged from 52 (0,0007%) to 11,233 (0,013%) per animal. Two mammarenavirus-positive animals were active (with IDs 22_15(MOS) and 23_1(MOW)), while the other two were hibernating (with IDs 22_7(MOS) and 22_6(BEL)). The high number of mammarenavirus reads in active animals was found in both oral and anal swab sequence data, providing indirect evidence that mammarenaviruses were replicating in the animals. The observation of mammarenavirus reads in hibernating animals supports the hypothesis that mammarenaviruses can replicate/persist for a long time in hedgehogs.

The arenaviruses have a two-segment genome: the large segment (L) of about 7,200 bp encodes the viral RNA-dependent RNA polymerase (*RdRp*) and a zinc-binding protein (ZP) and the small segment (S) of about 3,400 bp encodes the nucleocapsid protein (NP) and envelope glycoproteins (GP) (17, 54, 55). We were able to assemble a number of complete and partial L and S segments from several different mammarenaviruses designated as EriAreV (Erinaceus ArenaViruses) and deposited at NCBI (PQ041964- PQ041967, PQ059273, PQ059274, PQ059276- PQ059279). The annotations of assembled genomes are presented in Supplementary Table S12 “EriAreVs Assembly.”

The complete L and S segments of two closely-related mammarenavirus genomes were assembled using sequence data obtained from hedgehogs with IDs 22_7(MOS) and 23_1(MOW). The lengths of the assembled L segments were 7,325 and 7,354 nt, and the lengths of the S segments were 3,638 and 3,758 nt. Since the genetically related viruses MEMV and Alxa have nearly the same length of L and S segments of complete genomes from ICTV (in the range of 7,393–7,251 nt and 3,536–3,341 nt, respectively), we hypothesize that the assembled sequences are complete L and S segments. These viruses were designated EriAreV/RU/MOW/2023/hed23_1 and EriAreV/RU/MOS/2022/hed22_7 (short names ArV/23_1 and ArV/22_7). They showed 99.82–99.94% pairwise identity. Both animals in which these viruses were detected were caught in the city of Moscow and in the Moscow Region (in a small town).

In the two hedgehogs ID 22_15(MOS) and ID 22_6(BEL), the high SNP variability in the reads resulted in the assembly of several L and several S segments from one animal. The presence of multiple L and S arenavirus sequences was confirmed by assembly using different software (Spades and Genome Detective viral tool) and additional manual SNP checking. All assembled sequences were highly covered (average coverage 1961.17–2722.81). L and S segments of hedgehog with ID 22_6(BEL) showed 80.9% and 88,99% of nt pairwise identity The two variants of L and S segments of hedgehog ID 22_15(MOS) showed 72,8% and 94,23% of nt pairwise identity, respectively.

**Figure 7:**
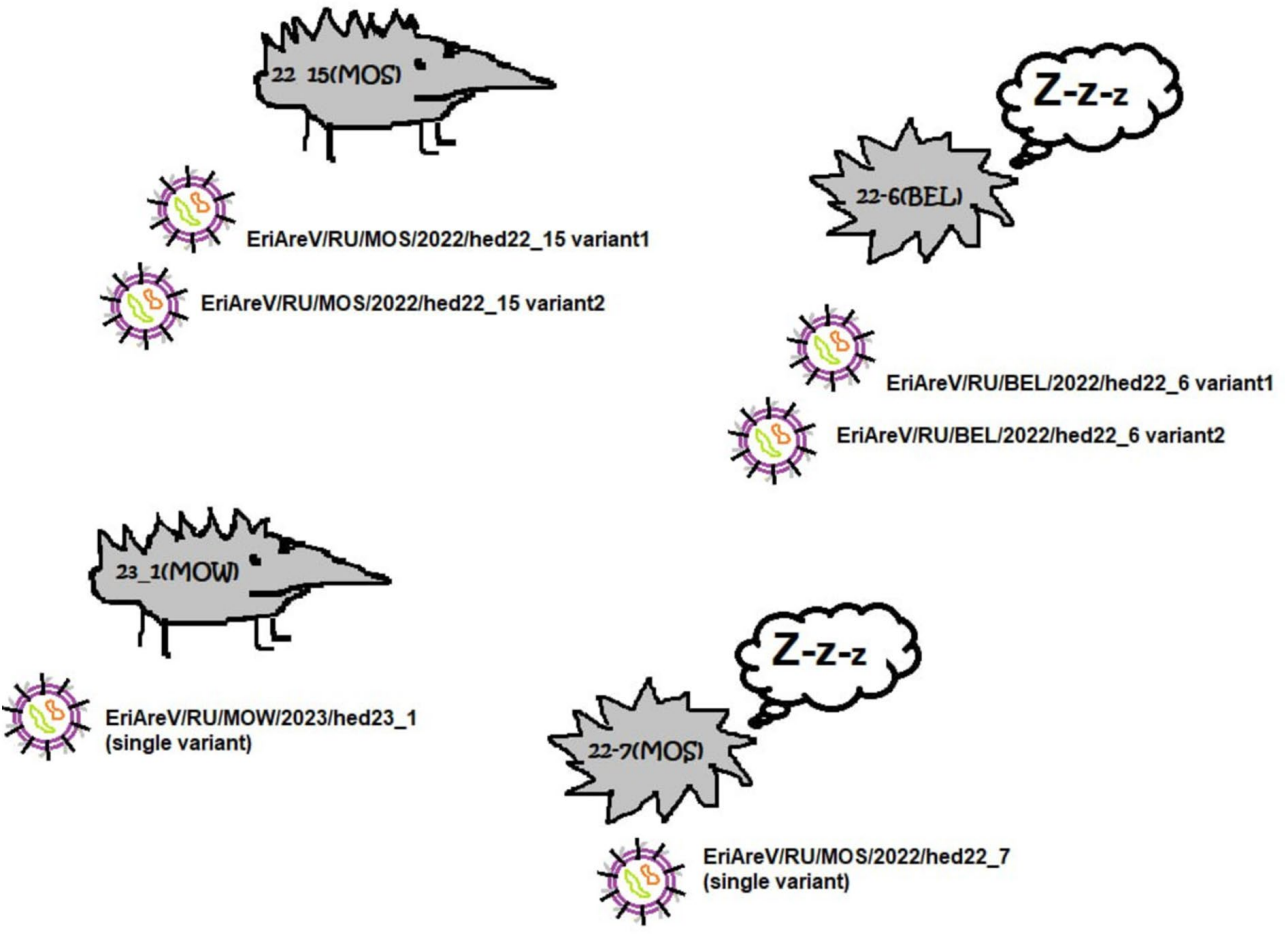
Mammarenavirus subvariants in the investigated animals.

Total, different subvariants of the L and S segments of the arenavirus genome were found in two of the four animals positive for EriAreV (one active and one hibernating) (Figure 7).

**Figure 8:**
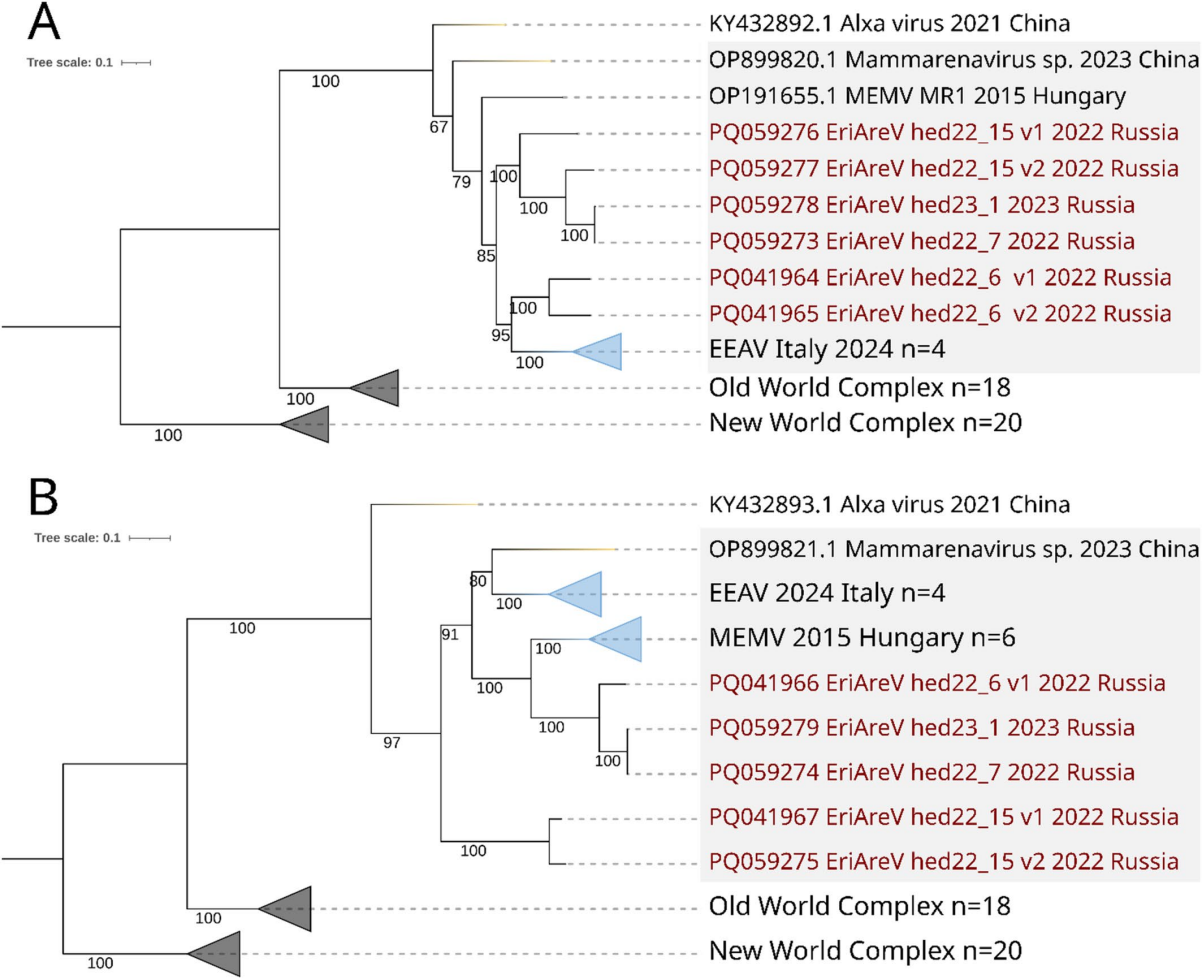
ML phylogenetic trees constructed for *Mammarenavirus* sequences. Mammarenaviruses from hedgehogs of different species are highlighted in grey. Best-fit model of substitution for both trees according to BIC: GTR + F + I + G4. Numbers indicate bootstrap values. Light purple stands are the Old-World complex, grey stands are the New World complex (viruses from different mammalian species). Mammarenaviruses assembled in this study are labelled red. **(A)** Tree constructed for 51 complete *RdRp* sequences (the most part of L segment); **(B)** Tree constructed for 55 complete *NP* (the part of S segment).

It should be noted that the variants of mammarenavirus segments from the same animal cluster together (Figure 8). According to the phylogenetic analysis, both L genomic segments found in one animal were closer to each other than to any other genome L segments. The same is true for S genomic segments found in the same specimen. Following the ICTV demarcation criterion, arenaviruses should be considered as potentially new species in the genus *Mammarenavirus* if they have less than 76% L and less than 80% S segment nucleotide sequence identity to any known member of the genus (56). In the two animals containing two different mammarenaviruses, the assembled genome segment sequences were so different that they definitely belong to different species. However, it makes us suggest that these subvariants are the result of the viral genome evolution during a prolonged infection of the animal. Presently, we can only conclude that the samples from these animals contain a complex of mammarenaviruses (subvariants), whose taxonomic position and origin could not be precisely determined.

Phylogenetic analysis showed that all EriAreVs from Russian *Erinaceus* sp. belong to the same clade as other mammarenaviruses from hedgehogs, while Alxa virus from Chinese *E. amurensis* and *Dipus sagitta* (family *Dipodidae*, its common name is the northern three-toed jerboa) was an outgroup. All together they form a clade distinct from all other Old World mammarenaviruses identified in various animals. We believe that the global circulation and diversification of hedgehog mammarenaviruses is taking place. No clear phylogeographic patterns have been revealed: trees constructed for sequences of *RdRp* (representing most of the L segment) and nucleoprotein (encoded by the S segment) show different patterns of relationships between viruses from Russia, Italy, Hungary, and China. At the same time, there is a pronounced clustering of related sequences by country, apparently reflecting the divergent evolution of the viruses.

### 3.4 Coinfection of multiple viruses

The sequencing of both oral and anal swabs from hedgehog ID 22-15(MOS) revealed an extremely high number of arenavirus and coronaviruses reads: 142054 (0.36%) and 145,604 (0.38%) of reads classified *as Coronaviridae* (*Betacoronavirus* genus) and 5,098 (0,013%) and 6,135 (0,016%) of reads classified as *Arenaviridae* (*Mammarenavirus* genus), respectively. There were also a few reads classified as *Picornaviridae*, 0.00008% in both oral and anal swabs. The presence of a large number of viral reads belonging to different viral families identified in both swabs (oral and anal) suggests that the animal was sick or immunosuppressed. However, it must be acknowledged that this is only a conjecture, as the animal in question was not subjected to a veterinary examination and it is therefore not possible to confirm with certainty that the animal showed symptoms of any disease. The complete genomes of two different betacoronaviruses and two different mammarenavirus strains were assembled from this animal. To test whether these results represent contamination from samples from other hedgehogs, we assembled genomes separately for oral swab and anal swab of animal ID 22-15(MOS). In both cases, we found the presence of reads belonging to two different virus genomes *Coronaviridae* (*Betacoronavirus* genus) and *Arenaviridae* (*Mammarenavirus* genus). This hedgehog “superspreader” was captured in a forest in the Moscow Region.

## 4 Discussion

In this section, we discuss the reasons for (1) the difference in viral composition found in three active and hibernated animals studied here, (2) the geographic distribution of hedgehog coronaviruses in the wild; (3) the unusual difference between two genomes of closely related betacoronaviruses from the same animal. (4) We discuss the possibility of arenaviruses chronical infection of hedgehogs, (5) geographic distribution of hedgehog arenaviruses in Europe, the genetic relationships of the found arenaviruses with their European relatives and (6) the need for further research and surveillance due to their potential threat to human health.

### 4.1 Difference in viral composition

The metavirome composition of fourteen active hedgehogs captured in European Russia (seven animals) and in Siberia (seven animals) has been described here. Additionally, the metaviromes of seven hibernated hedgehogs have been described firstly (all animals in hibernation were from European Russia). Differences in the viral composition of samples from hedgehogs from European Russia and central Siberia are most likely explained by the effect of chance and the distance between the two distant regions where samples were collected (about 2,800 kilometres between the Moscow region in European Russia and Novosibirsk in Siberia). We assume that some facts are most easily explained by local outbreaks (waves) of viral infection at the time of material collection, for example, the finding of parvoviruses in all samples from all animals from Siberia (and almost complete absence of parvoviruses in animals from European Russia). The same phenomenon was observed with plant viruses, for example, bromoviruses were found only in samples from animals from Siberia (and were completely absent in samples from animals from European Russia). Probably, some bromovirus infection was circulating among plants in Central European Russia at the time when swabs were taken from hedgehogs.

Identified read frequency and variability were extremely low in hibernating animals especially in comparison to those in active animals. We suspect that these are the effects of treatment in the rehabilitation center and lack of contact with natural virus carriers and each other. There were no traces of sickness or infection among the hibernating animals during visual inspection while sampling. The captive animals were kept in individual cages and never interacted with each other or with other animals. Another possible reason may be that the captive animals are fed a diet (that the diet of captive animals is different from that of their wild relatives).

### 4.2 Geographic distribution of hedgehog coronaviruses in the wild

We can conclude that betacoronaviruses are circulating in hedgehogs throughout all Europe, from west to east. Hedgehogs have been reported as natural reservoirs of coronaviruses since 2014 (9). Since then, in Western Europe the nucleic acids of coronaviruses have been registered many times in biological samples from hedgehogs (10, 11, 14, 16). Erinaceus coronaviruses represent a distinct clade within the phylogenetic tree of the genus *Betacoronavirus*. Until this report, the easternmost European location where EriCoVs were found in wild hedgehogs was in Poland (15). Our research clearly shows that viruses are present in hedgehogs in European Russia (Eastern Europe). There is an investigation described coronaviruses from Chinese hedgehogs (12, 13). In consideration of the aforementioned data, it can be supposed that hedgehogs across Eurasia should be considered as potential carriers of coronaviruses. Unfortunately, coronaviruses could not be detected in any of the hedgehogs from Siberia, however, it is probable that future studies will yield positive results. Regional differences in the prevalence of the hedgehog betacoronavirus have been observed by other researchers. In the UK, for example, hedgehogs positive for EriCoV were detected over a wide region of England and Wales, while no samples from Scotland tested positive (16). Like (16), we believe inferring population prevalence of viruses from restricted sampling is inappropriate due to potential for an accidental effect to influence the results. Coronaviruses in hedgehog populations from disparate geographic locations (but at approximately the same time) may be attributed to the occurrence of local outbreaks of these viruses.

The phylogenetic analysis has shown that betacoronaviruses from hedgehogs captured in western Europe (EriCoVs) are clearly distinct from Chinese betacoronaviruses (HKU strains) (see Figure 5). The two betacoronaviruses whose complete genomes were identified from hedgehogs captured in Moscow form a small clade between European and Chinese hedgehog betacoronaviruses. It is noteworthy that both betacoronaviruses from hedgehogs captured in Russia were closest to viruses from animals captured in distant locations, but not to viruses from the geographically closest location. This trend is also seen in the results of the phylogenetic analyses of both complete genomes and *RdRp* sequences. Erinaceus coronaviruses from Russia (2022) were genetically close to viruses from Italy (2018–2019), but not to geographically proximal Germany (2012) or Poland (2020). As hedgehogs are non-migratory animals, the presence of closer genetic relationships between viruses from the most geographically distant regions suggests that hedgehog coronaviruses are not only transmitted by hedgehogs but also by other, as yet unknown, means.

In the previous reports, there was no evidence to indicate that EriCoV was causing clinical disease in hedgehogs (16). It should be necessary to conduct experimental studies using cell lines to determine if EriCoVs bind to cell receptors in the tissues of hedgehogs. This should facilitate the estimation of the probability of EriCoV infection in these animals. Furthermore, a PCR diagnostic based on EriCoVs sequences could be used for analyzing clinical cases caused by an undetermined viral infection in animals.

### 4.3 An ancient recombination event may explain the difference between two genomes of closely related betacoronaviruses from the same animal

Hedgehog ID 22_15 (MOS) was a carrier of two coronaviruses. Comparative analysis of the genomes of these two betacoronaviruses suggested that one of them arose as a result of a recombination between two viruses, since all identified SNPs (n= 288) were found only in the local genome region. Recombination events have been recorded many times in the evolution of coronaviruses and they being considered as one of the driving forces of evolution behind host switching, especially when it happens in the spike gene (57–60). Many coronaviruses have an evolutionary history of recombination in the spike gene, including that of responsible for epidemics in domestic animals (61–65) and humans (66–69).

### 4.4 Possibility of a chronic infection of the hedgehog with arenaviruses

The presence of EriAreVs in captive hibernating *Erinaceus* sp. was surprising because these animals were kept under controlled conditions, ectoparasite-free, in separate cages, and had no contact with each other. The feature of known arenaviruses is their tendency to cause persistent infections in their natural hosts (rodents) as a result of a slow or inappropriate immune response (21, 70). Perharps, that mammarenaviruses entered in the hedgehogs we studied while they were foraging in the wild before they were captured (e.g., via rodents they ate) and then were replicated for a long time in the animals’ bodies. It is not known whether the wild-caught hedgehogs showed signs of disease. Anyway we suggest the possibility of persistent arenavirus infection in the hedgehogs. Further experiments may improve our knowledge of virus transmission, duration and the acute or asymptomatic character of arenavirus infection in hedgehogs.

Interestingly, more than one complete mammarenavirus genome segments (L and S) were successfully assembled from swabs obtained from animals with IDs 22_15(MOS) and 22_6(BEL). It should be noted that the assembly of two different segments of closely related mammarenaviruses from the same animals may not be sufficiently accurate. But the fact of presence of different viruses in the same animal is undoubted. The underlying cause of this phenomenon is unclear. Possible factors include long-term persistence of a single virus followed by evolution and the subspecies formation. Another possible explanation is the infection of animals with multiple mammarenaviruses, followed by recombination between homologous genomic segments and subsequent evolution. But animals with IDs 23_1(MOW) and 22_7(MOS) were infected by single mammarenavirus, suggesting that the genome sequences of EriAreV/RU/MOW/2023/hed23_1 and EriAreV/RU/MOS/2022/hed22_7 are indeed what viruses have in nature.

### 4.5 Geographic distribution of hedgehog arenaviruses in Europe and the genetic relationships of the found arenaviruses with their European relatives

Until 2023 there had been no evidence of European hedgehogs carrying mammarenaviruses, but it has been confirmed that hedgehogs are natural carriers of these viruses, at least since 2015 (18). In this study, we could assemble five complete genomes and one partial genome of new mammarenaviruses. The variability between genomes is substantial, therefore, according to the ICTV criteria, these viruses should be called different species (or the species criterion for *Mammarenavirus* should be changed). There is no reliable explanation why the obtained arenavirus genomes are so different, but this may be that either arenaviruses evolve quite quickly, or many genovariants circulate in nature. In any case, further research is required to confirm or refute the hypotheses. All in all, *Mammarenaviruses* do actually circulate in hedgehogs in the European part of Russia. Their epidemic potential is unclear and needs to be investigated as soon as possible. The reason for this is that *Mammarenavirus* infect rodents (55) and can be transmitted to humans, causing serious diseases (22, 71–73). As for hedgehogs, they occasionally come into contact with humans and are increasingly kept as pets. Due to this fact, it is of paramount importance to establish whether the identified hedgehog arenaviruses are able to infect humans or live-stock animals.

## 5 Conclusion

Our study shows that hedgehogs are carriers of coronaviruses and mammarenaviruses, and the latter most likely cause chronic disease in these animals. Importantly, hedgehogs are widely distributed in the countryside and cities where they live in close association with humans. The human (or domestic animal) pathogenicity of the currently identified hedgehog arenaviruses and coronaviruses is only a conjecture. Studies using laboratory cell lines as experimental models are needed to verify these assumptions. Diseases in hedgehogs caused by the identified coronaviruses and arenaviruses are possible (including chronic ones for arenaviruses). However, this statement also needs to be proven by molecular diagnosis of the pathogens in animals with clinical signs of disease. PCR diagnostic could be used for analyzing clinical cases caused by an undetermined viral infection in humans which were in contact with wild hedgehogs. Combined, these methods could assist in understanding the clinical significance of EriCoV infection in animals and possibility to affectihumans’ health.

## Data Availability

The datasets presented in this study can be found in online repositories. The names of the repository/repositories and accession number(s) can be found in the article/Supplementary material.

## Footnotes

^1^https://www.ncbi.nlm.nih.gov/orffinder/ (Accessed May 27, 2024)
