# Supplementary material for: Novel coronaviruses and mammarenaviruses of hedgehogs from Russia including the comparison of viral communities of hibernating and active specimens
Source: Front Vet Sci. 2024 Dec 16;11:1486635. doi: 10.3389/fvets.2024.1486635 (PMC11683907; doi:10.3389/fvets.2024.1486635)
Supplement: Supplementary file 2 [file Image_1.pdf]

Supplementary Material

Supplementary Figures

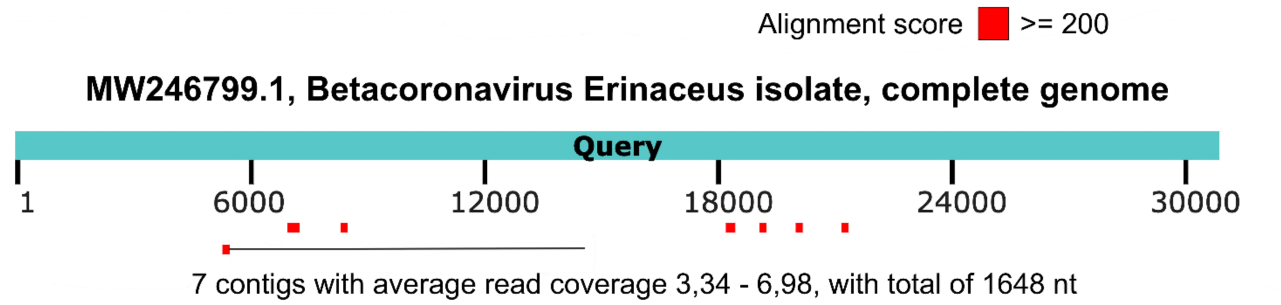

Supplementary Figure 1. The eight short contigs of 200-315 nt length with average read coverage of 4-10 from animal with ID 22_11(KRA) mapping to the reference genome (MW245799).
